# Exploring the interplay between gender, self-concept, motivation, and basic psychological needs among high school athletes

**DOI:** 10.3389/fpsyg.2026.1744182

**Published:** 2026-04-08

**Authors:** Arne Martin Jakobsen

**Affiliations:** Nord University, Bodø, Norway

**Keywords:** self-concept, self-determination, motivation, basic psychological needs, gender

## Abstract

This study investigates the relationship between gender and self-concept among high school students enrolled in sports-oriented programs. In addition, it examines the interconnections among self-concept, motivation, and the satisfaction of basic psychological needs. The sample comprises 215 high school students aged 16 to 18 years. Three standardized instruments were employed: the brief version of the Five-Factor Self-Concept Questionnaire, the Basic Psychological Needs in Exercise Scale, and the Sport Motivation Scale (SMS-II). Statistical analyses were conducted using IBM SPSS (version 28.0) and AMOS (version 29). As anticipated, no significant differences emerged in overall self-concept between male and female students, a finding consistent with prior research. However, a significant positive association was observed between gender and emotional self-concept, with female students scoring higher than their male counterparts. Regarding the relationship between self-concept and motivation, academic self-concept demonstrated a significant positive association with intrinsic motivation and a negative correlation with amotivation. Furthermore, academic self-concept was positively related to the basic psychological needs of autonomy, competence, and relatedness, with autonomy showing the strongest link. The study highlights the complex interplay among gender, self-concept, motivational orientations, and psychological needs. Emotional self-concept was positively correlated with less autonomous forms of motivation—introjected regulation, external regulation, and amotivation—suggesting a potential connection between emotional sensitivity and motivational vulnerability. Family self-concept emerged as a strong predictor of autonomous motivation, while social self-concept was significantly associated with the need for relatedness. In summary, the findings underscore the complex and multidimensional nature of self-concept and its significant implications for motivational processes.

## Introduction

The study is grounded in two complementary theoretical frameworks that together provide a robust foundation for understanding the relationships among self-concept, motivation, and basic psychological need satisfaction in sport-focused educational settings. The first is the five-factor model of self-concept proposed by Shavelson et al. (1976), which conceptualizes self-concept as a structured, hierarchical, and multidimensional construct. Within this model, self-concept is organized across both general and domain-specific levels, ranging from broad global self-perceptions to more discrete dimensions such as academic, social, physical, emotional, and competence-related domains. These dimensions are shaped through personal experiences and social feedback and can vary independently of one another, reflecting the flexible and context-sensitive nature of domain-specific self-beliefs. The hierarchical structure also implies that changes within a particular domain do not automatically translate to shifts in general self-concept, underscoring the importance of examining self-concept at multiple levels when studying adolescent development in sport environments (Shavelson et al., 1976; Marsh and Shavelson, 1985; Deci and Ryan, 1985). According to Marsh and Shavelson, “The self-concept organizes experiences and gives them meaning. It is our perceptions of ourselves. They are influenced by our evaluation from significant others, reinforcements, and attributes for our own behavior.” (p. 107) (Shavelson et al., 1976).

The second theoretical framework is Self-Determination Theory (SDT) developed by Deci and Ryan, a comprehensive model for understanding human motivation, personality development, and wellbeing (Deci and Ryan, 1985). Within SDT, particularly through the Basic Psychological Need Theory, motivation is understood as being driven by the satisfaction of three innate psychological needs: autonomy, the experience of volition and psychological freedom; competence, the perception of effectiveness and capability; and relatedness, the sense of connection and belonging with significant others (Ryan and Deci, 2017). When these needs are met, individuals are more likely to develop autonomous forms of motivation, experience wellbeing, and engage in behaviors that support long-term growth. Conversely, when these needs are thwarted, motivation becomes more controlled or amotivated, and wellbeing is compromised (Ryan and Deci, 2017).

Taken together, Shavelson’s model and Self-Determination Theory offer a coherent foundation for examining how students in sports-focused programs form perceptions of themselves and how these perceptions relate to their motivational functioning. The structured and multidimensional nature of self-concept aligns closely with SDT’s emphasis on competence and social context, suggesting that students’ self-beliefs, particularly in physical and academic domain are deeply connected to the degree to which their basic psychological needs are fulfilled. By integrating these two theoretical perspectives, the present study seeks to provide a nuanced understanding of how gender, self-concept, and motivational processes interact in shaping adolescents’ experiences within sport-oriented educational settings.

### Self-concept

As already mentioned self-concept encompasses multiple dimensions, including academic performance, social acceptance, physical attractiveness, and perceived competence. Its structure is hierarchical, with a general or global self-concept subdivided into academic and non-academic domains (Marsh and Shavelson, 1985; Garcia et al., 2018). The academic self-concept pertains to perceived competence across various school subjects—such as mathematics, history, and language—and reflects how capable individuals believe themselves to be in these areas (Alkhateeb et al., 2022; Steinberg et al., 2024). The non-academic self-concept comprises domains beyond the academic sphere and includes: Social self-concept, which is shaped by feedback from peers and significant others, and reflects perceived social acceptance and interpersonal relationships (Baron et al., 2014). Emotional self-concept, which is informed by emotional experiences and stress, and reflects perceived emotional stability and psychological wellbeing (Flores Kanter et al., 2015). Family self-concept, which reflects the subject’s perception of their involvement, participation, and integration in the family settings (Garcia et al., 2018) and last physical self-concept, which is based on perceptions of physical abilities and appearance, and reflects how individuals evaluate their physical characteristics and competencies (Shavelson et al., 1976; Marsh and Shavelson, 1985).

Each of these domains can be divided into more specific subdomains. For example, within academic self-concept, a student might have different perceptions of their abilities in various subjects like science, art, or physical education (Marsh and Shavelson, 1985).

General self-concept tends to remain relatively stable across time, even as the more specific dimensions that underpin it shift in response to everyday experiences. Changes within a particular domain whether academic, social, or physical do not automatically cascade upward to transform the broader sense of self. An athlete might revise their physical self-perceptions after a moment of success or failure, yet this adjustment often remains confined to the physical domain and does not necessarily reshape their overall global self-concept (Stake, 1992; Martinez-Marin et al., 2021). This dynamic reflects the hierarchical organization at the core of self-concept theory, in which domain-specific perceptions contribute to, but do not fully determine, the general self-view. Enhancements in a focused area such as mathematics may substantially strengthen a student’s academic self-concept within that subject, while leaving their global self-concept largely untouched. As individuals grow and accumulate a wider range of experiences, the structure of their self-concept becomes more nuanced and differentiated, allowing them to hold distinct evaluations of themselves across multiple spheres of life (Shavelson et al., 1976; Marsh and Shavelson, 1985).

These evaluations are constructed within situational contexts, where individuals draw simultaneously on personal ideals and on signals received from significant others. Internal benchmarks provide one frame of reference, yet perceptions of how peers, family members, and mentors appraise one’s abilities often exert a powerful influence (Shavelson et al., 1976; Marsh and Shavelson, 1985). The interplay between these absolute and relative standards contributes to the fluidity of self-concept, shaping how individuals interpret their competence and worth across diverse settings. Although self-concept shares conceptual space with constructs such as self-esteem, identity, and self-efficacy, it remains a theoretically distinct domain with its own patterns of development and expression. Research has shown that certain domains of self-concept align with gender-stereotypical expectations, particularly in areas linked to family or occupational roles. However, gender accounts for only a modest portion of the variability observed across these dimensions, indicating that contextual experiences, social environments, and individual histories are far more central in shaping how people come to understand themselves (Stake, 1992; Martinez-Marin et al., 2021; Gore and Cross, 2011).

Although both men and women often internalize traditional gendered self-concepts, these views do not remain static across the lifespan. As individuals mature, their understanding of gendered expectations tends to integrate more fully with their evolving sense of self, resulting in a generally more positive gendered self-concept. This gradual shift reflects increasing self-acceptance and a closer alignment between personal identity and the gender norms they have come to embrace over time (Martinez-Marin et al., 2021).

In certain domains, socially constructed gender roles may exert a greater influence on individual behavior and self-perception than biological sex. These roles are increasingly evolving toward androgyny, blending traditionally masculine and feminine traits across both male and female identities. This shift reflects a broader societal trend toward more fluid and inclusive understandings of gender, allowing individuals to express a wider range of characteristics irrespective of their biological sex (Martinez-Marin et al., 2021; Marcic and Kobal Grum, 2011; Waghmare, 2018).

In a study conducted by Waghmare (2018), female students reported significantly higher levels of physical and social self-concept compared to their male counterparts. In contrast, male students exhibited higher scores in educational, moral, and intellectual self-concept domains. Nonetheless, the study found no statistically significant difference in overall self-concept between male and female college students (Waghmare, 2018).

In their meta-analysis, Wilgenbusch and Merell (1999) found that boys in both elementary and secondary education exhibited significantly higher self-concept in the domains of physical appearance and athletic/psychomotor coordination compared to girls.

### Motivation

Self-Determination Theory (SDT) constitutes a comprehensive framework for understanding human motivation and personality development (Deci and Ryan, 1985; Ryan and Deci, 2017). It examines the extent to which human behavior is self-motivated and self-determined. SDT is composed of six interrelated mini-theories, each addressing specific dimensions of motivation.

This study draws on Basic Psychological Need Theory (BPNT), a central sub-theory within Self-Determination Theory (SDT) that specifies the conditions required for human motivation and wellbeing (Vansteenkiste et al., 2023, 2020). SDT provides the overarching framework, proposing that individuals are naturally inclined toward growth, internalization, and self-regulation, but that these processes depend on the extent to which the social environment supports three basic psychological needs. BPNT elaborates on this foundation by identifying autonomy, competence, and relatedness as the essential nutrients for psychological functioning (Vansteenkiste et al., 2023; Vlachopoulos et al., 2011). Autonomy concerns the experience of volition and self-endorsement in one’s actions; competence reflects the felt sense of effectiveness and mastery within a given context; and relatedness captures the need to feel meaningfully connected to others, both in giving and receiving care (Vansteenkiste et al., 2004; Ryan and Deci, 2020).

Within this theoretical structure, the satisfaction of basic psychological needs is understood as the primary mechanism through which high-quality motivation and wellbeing emerge. When individuals feel autonomous, competent, and socially connected, they are more likely to engage in activities with intrinsic motivation and to exhibit adaptive patterns of functioning, such as persistence, enjoyment, and psychological vitality (Ryan and Deci, 2020; Vansteenkiste et al., 2007). Conversely, when these needs are frustrated through controlling environments, repeated failure experiences, or social exclusion motivation becomes more controlled or amotivated, and individuals are at heightened risk for maladjustment (Ratelle et al., 2007; Ames and Archer, 1988). In sport contexts, need frustration has been consistently linked to negative outcomes, including emotional exhaustion, disengagement, and reduced wellbeing. BPNT therefore emphasizes that the degree to which basic needs are supported or thwarted within a given environment not only shapes athletes’ motivational quality but also their broader psychological adjustment, making it a particularly relevant framework for examining motivational processes in youth sport settings (Vansteenkiste et al., 2023, 2020; Wang and Hagger, 2023).

Within Self-Determination Theory (SDT), motivation is conceptualized along a continuum that includes intrinsic motivation, extrinsic motivation, and amotivation. Intrinsic motivation refers to engaging in an activity for the inherent satisfaction and enjoyment it provides. Extrinsic motivation refers to behavior that is performed as a means to achieve outcomes that lie outside the behavior itself (Ryan and Deci, 2020). Amotivation represents a state of lacking intentionality or motivation to act. This condition arises when individuals perceive no meaningful connection between their actions and outcomes, feel ineffective or incompetent, or regard the activity as irrelevant to their psychological needs (Ryan and Deci, 2019).

Intrinsic motivation is considered more beneficial for long-term engagement and wellbeing. SDT categorizes extrinsic motivation into four types, based on the degree of autonomy. With the lowest degree of autonomy, we have external regulation which is behavior driven by external rewards or punishments (Ryan and Deci, 2020). This is followed by introjected regulation which is behavior driven by internal pressures, such as guilt or anxiety. A more autonomous behavior is identified regulation which is driven by personal goals and values. Integrated regulation is considered the most autonomous form of external motivation, where behavior is fully aligned with one’s personal values and needs (Ryan and Deci, 2017, 2019).

Understanding the different forms of extrinsic regulation within SDT is crucial for designing environments that foster more autonomous forms of motivation, which in turn contribute to greater engagement, wellbeing, and sustained personal development (Deci and Ryan, 2002; Vansteenkiste and Deci, 2003; Wilson et al., 2003).

Several studies have examined the relationship between self-concept and motivation. For instance, a study conducted in Turkey found a positive association between self-concept clarity and intrinsic motivation, suggesting that individuals with a clearer and more stable sense of self are more likely to engage in activities driven by inherent interest and personal satisfaction (Özcan et al., 2025). This finding supports the notion that a well-defined self-concept may serve as a foundation for autonomous motivation and psychological wellbeing (Özcan et al., 2025). Further research has demonstrated a direct link between self-concept and academic achievement, with intrinsic motivation serving as a significant mediating factor. For example, Khalaila (2015) found that individuals with higher levels of self-concept tend to perform better academically, and this relationship is significantly mediated by intrinsic motivation. Moreover, studies have identified a positive correlation between academic self-concept and the three basic psychological needs autonomy, competence, and relatedness with autonomy and competence emerging as particularly salient among male students (Chacon-Cuberos et al., 2025). Basic psychological needs have also been shown to be significantly interrelated, and they demonstrate a significant positive association with physical self-concept among adolescents (Fraguela-Vale et al., 2020).

This study examines the influence of gender on self-concept among high school students enrolled in sports-specialized educational programs and investigates how different dimensions of self-concept relate to students’ motivational orientations and the fulfillment of basic psychological needs. Drawing on the theoretical framework presented above, as well as the meta-analysis by Wilgenbusch and Merell (1999), which found that boys report significantly higher self-concept in the domains of physical appearance and athletic or psychomotor competence compared to girls, the following hypotheses are proposed:

### Hypotheses

Gender differences in self-concept:

*H1*: Male high school students specializing in sports will have higher physical self-concept than female students.

*H2*: There will be no significant difference between male and female high school students in overall self-concept.

Relationship between self-concept and motivation:

*H3*: Students with higher physical self-concept will show higher levels of intrinsic motivation towards sports activities.

Basic psychological needs and self-concept:

*H4*: Satisfaction of basic psychological needs (autonomy, competence, and relatedness) will positively correlate with higher self-concept in various domains (academic, social, emotional, physical and family).

## Materials and methods

### Participants

The sample consisted of 215 Norwegian high school students specializing in sports, aged between 16 and 18 years (M = 17.04, SD = 0.83). The group included 115 boys and 100 girls. The programs consist of both theoretical and practical subjects. Inclusion criteria required that all participants be enrolled in a sports specialization program. The study was approved by the Norwegian Agency for Shared Services in Education and Research (Sikt). Parental consent was obtained for participants under 18 years of age.

### Procedure

Data collection was conducted during a scheduled school hour, with one of the researchers present to oversee the process. The questionnaires were administered via Google Docs, ensuring a standardized and accessible format for all participants. Data is not registered with email addresses. It is not possible to identify the students. Four students were absent during the data collection session. Upon completion, the data were transferred to SPSS for statistical analysis and further processed using both SPSS and AMOS software. Gender is coded boys 1 and girls 2.

### Instruments

For the purposes of this study, all instruments were translated into Norwegian and subsequently back-translated into English by an English teacher to ensure linguistic and conceptual equivalence. The Norwegian versions of the scales were validated using confirmatory factor analysis (CFA) in AMOS and were also pilot-tested on a sample of students to assess clarity and reliability. The scale was tested on a pilot group consistent of 22 bachelor students in sport.

### Five-factor self-concept (AF5)

The brief version of the Five-Factor Self-Concept Questionnaire (AF5) (Grau et al., 2014) was developed to assess self-concept across five distinct dimensions, offering a psychometrically robust and multidimensional approach to measuring self-concept. The instrument comprises five subscales, each consisting of four items: Academic self-concept (e.g., “My teachers think I am a good student”; *α* = 0.86), social self-concept (e.g., “I have a lot of friends”; *α* = 0.77), emotional self-concept (e.g., reverse-scored “I feel nervous”; *α* = 0.83), family self-concept (e.g., “I feel happy at home”; *α* = 0.84), physical self-concept (e.g., “I am an attractive person”; *α* = 0.80).

All subscales demonstrated satisfactory internal consistency, with Cronbach’s alpha values ranging from acceptable to high. Participants responded using a seven-point Likert scale, reflecting the degree to which each statement applied to them. While all factors showed good reliability, the social self-concept subscale was deemed acceptable. A confirmatory factor analysis (CFA) was conducted, yielding acceptable model fit indices: NFI = 0.87, RMSEA = 0.08, CFI = 0.92, indicating that the five-factor structure was appropriately supported by the data.

### Basic psychological needs

The Basic Psychological Needs in Exercise Scale (BPNES) was employed to assess students’ satisfaction with their basic psychological needs. The instrument comprises three subscales—autonomy, competence, and relatedness—each consisting of four items. This structure allows for a focused evaluation of the extent to which students feel volitional, effective, and socially connected in their exercise and sport-related activities (Vlachopoulos et al., 2011, 2010). To complete the Basic Psychological Needs in Exercise Scale (BPNES), students responded to a series of statements using a seven-point Likert scale, where 1 indicated “does not agree at all” and 7 indicated “completely agrees.” The scale assessed satisfaction with three core psychological needs: Autonomy (e.g., “The way I train is clearly an expression of how I want the training to be”; *α* = 0.81), competence (e.g., “I feel I can handle the tasks the training sets for me”; *α* = 0.78), relatedness (e.g., “I feel like I can trust the others I train with”; *α* = 0.86).

All subscales demonstrated acceptable to good internal consistency, with competence showing acceptable reliability and autonomy and relatedness showing good reliability. The BPNES has been validated in several studies (Vlachopoulos et al., 2010; Erdvik et al., 2019). A confirmatory factor analysis (CFA) was conducted, yielding model fit indices of NFI = 0.90, RMSEA = 0.09, CFI = 0.94, indicating an appropriate fit between the model and the data.

### Sport motivation scale II (SMS II)

The revised version of the Sport Motivation Scale (SMS-II) (Pelletier et al., 2013) was used to assess students’ motivation in sport and physical activity contexts (Pelletier and Sarrazin, 2007; Mallett et al., 2007). The scale comprises six motivational factors, each represented by three items, with responses rated on a seven-point Likert scale ranging from 1 (“not correct at all”) to 7 (“completely correct”) (Lavrakas, 2008). The six factors include: Intrinsic regulation (e.g., “Because it is very interesting to learn how I can improve”; *α* = 0.77), integrated regulation (e.g., “Because practicing sports reflects the essence of who I am”; *α* = 0.72), identified regulation (e.g., “Because I found it is a good way to develop aspects of myself that I value”; *α* = 0.76), introjected regulation (e.g., “Because I would feel bad about myself if I did not take the time to do it”; *α* = 0.53), external regulation (e.g., “Because people I care about would be upset with me if I did not”; *α* = 0.52), non-regulation (amotivation) (e.g., “I do not know anymore; I have the impression that I am incapable of succeeding in this sport”; *α* = 0.70).

While most subscales demonstrated acceptable internal consistency, the reliability of Introjected Regulation and External Regulation was poor. A confirmatory factor analysis (CFA) was conducted to evaluate the scale’s structural validity, yielding satisfactory model fit indices: NFI = 0.92, RMSEA = 0.02, CFI = 0.99, indicating a strong fit between the model and the data (Hair et al., 2018).

### Data analysis

All statistical analyses were conducted using IBM SPSS (version 28.0) and AMOS (version 29). The validity of the scales was analyzed via a confirmatory factor analysis (CFA)(AMOS), and Cronbach’s alpha was employed to assess the internal reliability of each scale. Descriptive statistics and bivariate correlations for the self-concept and for the motivation were calculated.

**Figure 1:**
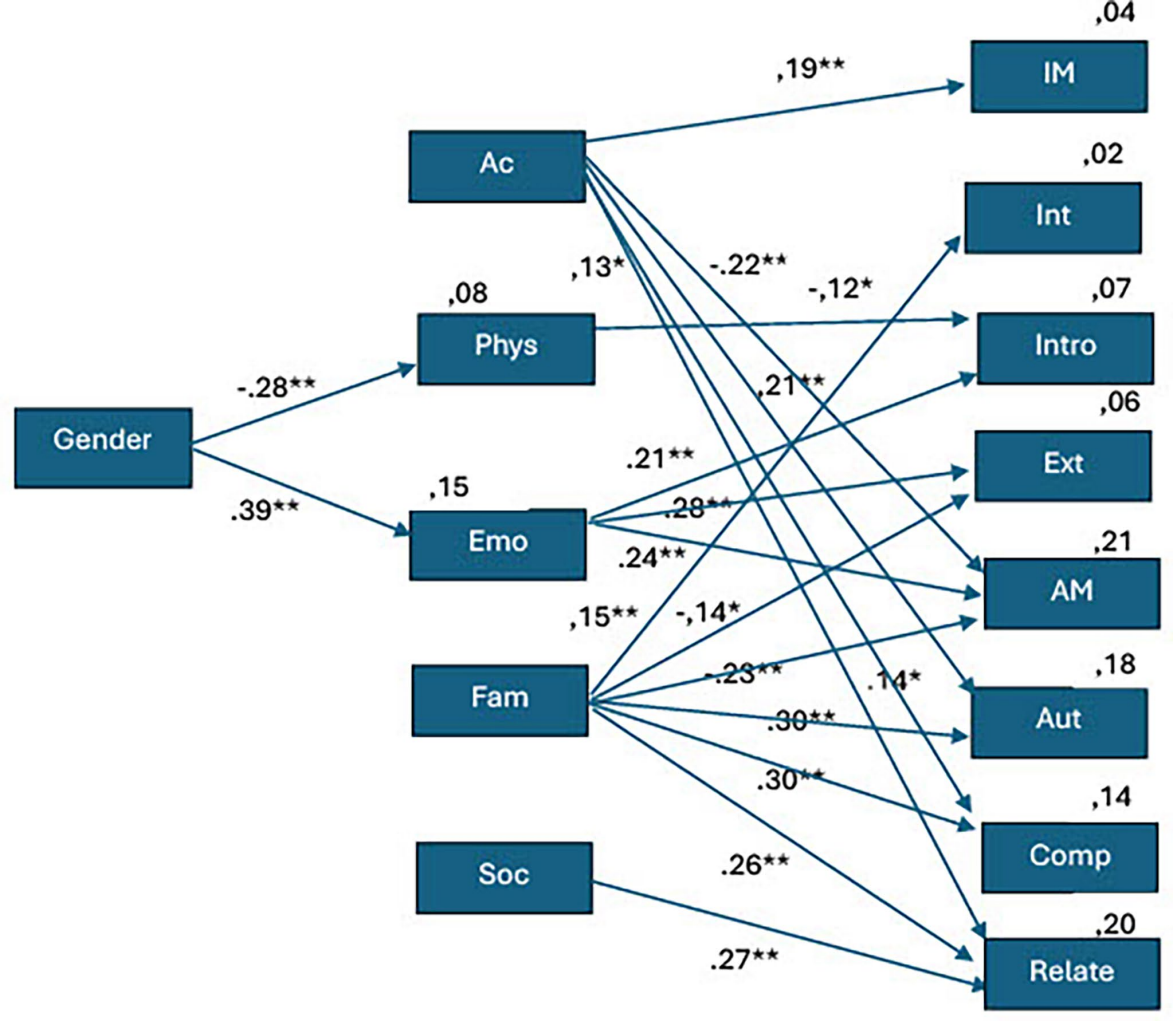
The modified hypothesized SEM model. Ac = academic self-concept, Phys = physical self-concept, Emo = emotional self-concept, Fam = family self-concept, Soc = social self-concept, IM = intrinsic motivation, Int-integrated regulation, Intro = introjected regulation, Ext = extrinsic regulation, AM = amotivation, Aut = need for autonomy, Comp = need for competence, Relate = need for relationship Chi-square = 35.5, probability level = 0.265 model fit TLI = 0.988, CFI = 0.996, and RMSEA = 0.026. *p < 0.05, **p < 0.01.

The originally hypothesized model was tested and modified with the use of structural equation modeling (AMOS). Global fit was evaluated using *χ*^2^, CFI, TLI, RMSEA (90% CI), and SRMR. Local misfit was inspected via modification indices (MIs) with a reporting threshold of MI ≥ 10 and the associated expected parameter change (EPC). Each eligible change was introduced singly, followed by re-estimation and re-assessment of fit. Final decisions were summarized in a dedicated MI tabl. The final best-fitting model for the sample is shown in Figure 1. The chi-square for the model was 35.5 with a probability level of 0.265. The CFI = 0.996, TLI = 0.988 and RMSEA = 0.026 indicated a good fit of the model.

Preliminary screening of skewness and kurtosis revealed no significant violations of normality, supporting the suitability of the data for subsequent SEM analysis.

## Results

### Scale analyses

Descriptive statistics and bivariate Pearson correlations were calculated for all subdimensions of intrinsic and extrinsic motivation, as well as for the three basic psychological needs, autonomy, competence, and relatedness. These results are presented in Table 1 and provide an overview of central tendencies, variability, and the interrelationships among the key constructs in the model.

**Table 1:** Correlation between all motivational regulations and psychological basic needs.

	IM	Intreg	Idreg	Introjr	Extreg	AM	Aut	Comp	Relate
IM	1								
Intreg	0.54^**^	1							
Idreg	0.58^**^	0.72^**^	1						
Introjreg	0.20^**^	0.40^**^	0.32^**^	1					
Exrge	0.05	0.22^**^	0.13	0.29^**^	1				
Amot	−0.31^**^	−0.32^**^	−0.29^**^	−0.14^*^	0.22^**^	1			
Aut	0.48^**^	0.43^**^	0.43^**^	0.19^**^	−0.09	−0.46^**^	1		
Comp	0.41^**^	0.43^**^	0.40^**^	0.22^**^	−0.03	−0.43^**^	80^**^	1	
Relate	0.28^**^	0.29^**^	0.30^**^	0.10	−0.05	−0.40^**^	0.69^**^	0.72^**^	1
Mean	5.47	5.65	5.59	5.59	3.98	2.10	5.66	5.45	5.68
Std. d	0.49	0.95	1.02	1.03	1.18	1.01	0.96	0.91	1.07
*n*	215	215	215	215	215	215	215	215	215
*α*	0.77	0.72	0.76	0.53	0.52	0.70	0.81	0.78	0.86

Including: mean, standard deviation, population (*n*) and chrombachs alpha (*α*). *p < 0.05, **p < 0.01.

The students demonstrated high mean scores across all motivational dimensions, including intrinsic motivation, integrated regulation, identified regulation, and introjected regulation. No statistically significant differences were observed between these mean scores within the sample.

Descriptive statistics and bivariate correlations for self-concept and gender were also computed (see Table 2). A significant moderate positive correlation was found between gender and emotional self-concept (*p* < 0.01, *r* = −0.37). Conversely, a significant moderate negative correlation was observed between gender and physical self-concept (*p* < 0.01, *r* = −0.33).

**Table 2:** Correlation between gender, academic-, social-, emotional-, family-, physical- and overall self-concept.

	Gender	Academic SC	Social SC	Emotional SC	Fam SC	Physical SC	Overall SC
Gender	1						
Academic SC	0.05	1					
Social SC	−0.11	0.36**	1				
Emotional SC	0.37**	0.03	0.05	1			
Family SC	−0.09	0.39**	0.56**	−0.12	1		
Physical SC	−0.33**	0.38**	0.49**	−0.33**	0.57**	1	
Overall SC	0.02	0.71**	0.71**	0.32**	0.72**	0.62**	1
Mean		5.27	4.90	3.29	5.95	4.90	4.86
Std. d		1.17	0.65	1.44	1.03	1.11	0.64
*N*	215	215	215	215	215	215	215
*α*		0.86	0.77	0.83	0.84	0.80	0.78

Including: mean, standard deviation, population (*n*) and Chronbachs alpha (*α*). *p < 0.05, **p < 0.01.

The students reported high mean scores across multiple self-concept dimensions, including academic, social, and family self-concept. These constructs were significantly and positively interrelated (*p* < 0.01). In contrast, emotional self-concept emerged as the only dimension with a comparatively low mean score. Moreover, emotional self-concept exhibited a statistically moderate significant negative correlation with physical self-concept (*p* < 0.01, *r* = −0.33). Despite this inverse relationship, the overall self-concept score was notably high (M = 4.86).

### Gender and self-concept

This study utilized Structural Equation Modeling (SEM) to examine the complex interrelations among gender, self-concept, motivation, and basic psychological needs. The analytical model incorporated only those variables that demonstrated statistically significant direct or indirect effects. As previously noted, the model exhibited satisfactory fit indices, indicating its appropriateness for the data. Identified regulation, which represents the most autonomous form of extrinsic motivation, showed no significant associations with any of the five self-concept dimensions and was therefore excluded from the figure. Multicollinearity was assessed in SPSS prior to SEM. All predictors showed acceptable collinearity levels (VIF < 5; tolerance > 0.20). All factors demonstrated acceptable levels of univariate normality, with skewness values falling within the ±2 range and kurtosis values within the ±3 range.

Figure 1 illustrates that gender exerted a statistically significant influence on two dimensions of self-concept. Specifically, a moderate negative association was observed between gender and physical self-concept (*R*^2^ = 0.08) (*p* < 0.01, *β* = −0.28). Conversely, a moderate positive relationship was identified between gender and emotional self-concept (*R*^2^ = 0.15) (*p* < 0.01, *β* = 0.39).

### Self-concept and motivation

Among the various dimensions of self-concept, only academic self-concept was found to significantly predict intrinsic motivation (*R*^2^ = 0.04) (*p* < 0.01, *β* = 0.19).

Furthermore, integrated regulation, recognized as the most autonomous form of extrinsic motivation, was exclusively influenced by familiar self-concept (*R*^2^ = 0.02) (*p* < 0.01, *β* = 0.15).

None of the self-concept dimensions exhibited a statistically significant relationship with identified regulation. However, introjected regulation (*R*^2^ = 0.07), considered a relatively controlled form of motivational regulation, was found to be negatively associated with physical self-concept (*p* < 0.05, *β* = −0.12) and positively associated with emotion implications and al self-concept (*p* < 0.01, *β* = 0.21).

Emotional self-concept also had a positive relationship with the least autonomous motive; extrinsic regulation (*R*^2^ = 0.06) (*p* < 0.01, *β* = 0.28) and amotivation (*p* < 0.01, *β* = 0.24). Those with a low score on both extrinsic regulations and amotivation (*R*^2^ = 0.21) also had a low score on emotional self-concept.

### Self-concept and basic psychological needs

The basic psychological need for autonomy (*R*^2^ = 0.18) demonstrated a statistically significant positive association with academic self-concept (*p* < 0.01, *β* = 0.21). Similarly, autonomy was positively correlated with family self-concept (*p* < 0.01, *β* = 0.30).

The second basic psychological need competence (*R*^2^ = 0.14) was positively associated with both academic(*p* < 0.05, *β* = 0.14) and family (*p* < 0.01, *β* = 0.30) self-concept.

The third basic psychological need relatedness (*R*^2^ = 0.20) was positively associated with three dimensions of self-concept. Specifically, the relationship with academic self-concept was statistically significant at the 5% level (*p* < 0.05, *β* = 0.13), while the associations with family and social self-concept were significant at the 1% level (*p* < 0.01, *β* = 0.26). Likewise, students with a positive family self-concept and a strong social self-concept are more likely to experience moderate fulfilling and meaningful social interactions (*p* < 0.01, *β* = 0.27).

## Discussion

An empirical investigation was conducted into the influence of gender on self-concept among high school students enrolled in sport-specialized educational programs. Furthermore, the associations between distinct dimensions of self-concept and students’ motivational orientations both intrinsic and extrinsic were examined, along with the extent to which these dimensions relate to the satisfaction of basic psychological needs, specifically autonomy, competence, and relatedness.

### Gender differences in self-concept

Grounded in Self-Determination Theory (Deci and Ryan, 1985) and existing research on self-concept and academic motivation, our study hypothesized that male high school students specializing in sports would exhibit a higher physical self-concept than their female counterparts, while no significant gender differences would be found in overall self-concept. Additionally, the analysis explored whether students with a stronger physical self-concept demonstrate higher levels of intrinsic motivation toward sports activities.

The data suggest that male students generally report higher levels of physical self-concept, indicating that female students tend to perceive their physical abilities and attributes less positively than their male counterparts. However, this pattern contrasts with results from other studies, which have found that female students display higher levels of both physical and social self-concept than male students (Waghmare, 2018).

A significant positive association was also observed between gender and emotional self-concept, indicating that female students scored higher than male students on this dimension. These findings are consistent with Wilgenbusch and Merell (1999), who reported that boys in secondary school possessed a more pronounced physical self-concept compared to their female peers. The results also align with research suggesting that empathy and emotionally oriented traits are shaped by contextual influences and may be systematically affected by gender roles and stereotypical expectations (Löffler and Greitemeyer, 2021). Furthermore, emotional attention has been identified as an important predictor of gendered self-concept, implying that individual differences in the capacity to attend to emotional states may contribute to systematic variation in self-concept across genders (Martinez-Marin et al., 2021).

As anticipated, no significant differences were observed in overall self-concept between male and female students. This finding is consistent with previous research that has reported similar results (Waghmare, 2018).

These two hypothesis is then confirmed.

### Self-concept and motivation

In examining the relationship between self-concept and motivation, we found that academic self-concept was significantly and positively associated with intrinsic motivation Additionally, academic self-concept demonstrated a negative correlation with amotivation. This finding supports previous research demonstrating that academic self-concept is associated with academic achievement, either directly or indirectly through the mediating role of intrinsic motivation (Khalaila, 2015).

Previous studies has found out that intrinsic motivation mediates the relationship between physical self-concept and physical performance (Lohbeck et al., 2021).

Furthermore physical self-concept is significantly associated only with introjected regulation, which is considered one of the least autonomous forms of motivational regulation. This relationship is negative indicating that individuals with a high physical self-concept tend to exhibit lower levels of introjected regulation rather than by autonomous endorsement of the activity (Ryan and Deci, 2019). In other words, they are less influenced by external pressures such as the need to avoid disapproval or to gain approval from others (Ryan and Deci, 2017, 2019). Because the item demonstrated low reliability (*α* = 0.53), this relationship should be interpreted with caution and not given substantial weight in the overall analysis. As previously observed, male students report a higher physical self-concept compared to female students. While this can generally be regarded as a positive outcome, it contrasts with our initial expectations (Fraguela-Vale et al., 2020). This expectation is supported by research showing that children who are intrinsically motivated in physical activities demonstrate higher motor skill performance when their physical self-concept is strong. Children who perceive themselves as competent in physical activities tend to perform better when they are intrinsically motivated, that is, when they engage in physical activity for its inherent enjoyment rather than in response to external pressure (Lohbeck et al., 2021).

The absence of such a relationship suggests a more complex dynamic between self-perception and motivational processes than initially anticipated (Martin-Albo et al., 2012). Previous research indicates that physical activity among male students is predominantly predicted by intrinsic motivation, identified regulation, and introjected regulation. This pattern suggests that boys are motivated to engage in physical activity through a combination of autonomous factors such as enjoyment and personal importance and controlled factors, including internal pressure and the pursuit of external approval. In contrast, for female students, identified regulation stands out as the sole significant predictor. This implies that girls are primarily driven by self-endorsed values and goals, rather than by external contingencies (Pavlovic et al., 2023). Our hypothesis that students with a higher physical self-concept would exhibit greater intrinsic motivation toward sports activities must be rejected. The data did not support a positive relationship between physical self-concept and intrinsic motivation. However, our findings did show that students with a high physical self-concept tended to score lower on introjected regulation, suggesting they are less driven by internal pressures such as guilt or obligation.

Previous findings indicate that female students scored higher on emotional self-concept compared to their male counterparts. Emotional self-concept is significantly and positively correlated with introjected regulation, external regulation, and amotivation, all of which are characterized by low levels of autonomy. This association suggests that individuals with elevated emotional self-concept scores also tend to exhibit higher levels of these less autonomous forms of motivation. Emotional self-concept is grounded in an individual’s emotional experiences and stress responses, encapsulating their perceived emotional stability and overall psychological wellbeing (Flores Kanter et al., 2015). It reflects how individuals interpret and manage their emotional states in various social contexts. For example, one item used to measure emotional self-concept reads: “When older people say something to me, I get very nervous,” illustrating the role of interpersonal interactions in shaping emotional self-perception.

Family self-concept demonstrates a significant positive association with integrated regulation, a form of autonomous extrinsic motivation (Ryan and Deci, 2019, 2000).

Family self-concept also showed a significant negative correlation with extrinsic regulation, indicating that individuals who perceive strong support and cohesion within their families are less influenced by external rewards or pressures. Because this item also demonstrated low reliability (*α* = 0.52), this relationship should be interpreted with caution and not given substantial weight in the overall analysis. Prior research similarly highlights the role of the family environment in shaping children’s motivation and participation in sport. Studies have shown that parents’ sport-related socialization behaviors exert both direct and indirect effects on children’s engagement in organized after-school sports activities. More specifically, children’s self-concept of ability and their interest in sport have been found to mediate the relationship between parental behaviors and participation levels (Jiang et al., 2025).

The hypothesis proposing that students with higher physical self-concept would exhibit higher levels of intrinsic motivation toward sports activities must therefore be rejected.

### Self-concept and basic psychological needs

In this study only academic- and family self-concept showed statistically significant relationship with all three basic psychological needs. Social self-concept only showed relationship with the basic need relatedness.

Academic self-concept was positively related to all three basic psychological needs. The strongest association was observed with autonomy, while competence and relatedness also showed statistically significant relationships. These findings find support in earlier studies which have found a positive association between different dimensions of self-concept and basic psychological needs in higher education (Chacon-Cuberos et al., 2025; Fraguela-Vale et al., 2020).

Moreover, family self-concept is strongly and positively associated with all three basic psychological needs; autonomy, competence, and relatedness indicating that those who perceive strong familial support and connection are more likely to experience fulfillment in these foundational aspects of motivation and wellbeing. Empirical research has demonstrated a significant relationship between parental autonomy support, the fulfillment of basic psychological needs, and the development of children’s self-concept (Chen et al., 2024). Basic psychological needs; autonomy, competence, and relatedness serve as partial mediators in the link between parental autonomy support and children’s self-concept (Nocito et al., 2020; Johansen et al., 2023). Notably, the influence of autonomy support varies depending on the specific need type, whereas parental control consistently exerts a negative predictive effect. The pathways through which autonomy support and control shape self-concept differ across the three psychological needs, with variations observed by gender and age. Specifically, boys and older children exhibit stronger associations with competence needs, while girls show heightened sensitivity to autonomy needs. Both groups, however, demonstrate responsiveness to relatedness needs (Chen et al., 2024).

In this study social self-concept is significantly and positively associated only with the basic psychological need of relatedness. This suggests that individuals who perceive themselves as socially competent or connected tend to experience greater satisfaction in feeling understood, cared for, and emotionally bonded with others. Previous studies have demonstrated that, at the within-subjects level, relatedness satisfaction significantly mediates the relationship between technology-based interactions and overall wellbeing. In contrast, autonomy satisfaction defined as the sense of self-initiation and ownership over one’s decisions and behaviors has been found to mediate the relationship between face-to-face interactions and wellbeing at the within-person level (Dimmock et al., 2021).

Physical and emotional self-concept did not show any significant associations with any of the basic psychological needs. Previous studies, however, have reported positive relationships between psychological needs and physical self-concept (Fraguela-Vale et al., 2020). The absence of such associations in the present study is difficult to account for and may reflect contextual, methodological, or sample-specific factors.

The hypothesis proposed that satisfaction of the basic psychological needs autonomy, competence, and relatedness would be positively associated with higher self-concept across multiple domains (academic, social, emotional, physical, and family). This assumption is only partially supported by the findings.

### Implications

A potential implication of these findings is that teachers and coaches working with young athletes may benefit from placing greater emphasis on supporting students’ academic and family self-concept. Strengthening these dimensions may help students develop a more stable and positive sense of personal competence and belonging across different life domains. Such experiences can generalize into the sport context, where young athletes who feel supported academically and at home may be more likely to internalize their participation, experience activities as meaningful, and maintain higher levels of intrinsic motivation.

By fostering these aspects of self-concept, practitioners may also contribute to greater satisfaction of students’ basic psychological needs for autonomy, competence, and relatedness. When these needs are met consistently across school, family, and sport, young athletes are more likely to show adaptive motivational patterns, including greater enjoyment, stronger engagement, and more self-regulated forms of motivation. Ultimately, this could enhance persistence and reduce dropout rates by creating an environment where students feel supported as whole individuals not only as athletes, but also as learners and family members whose broader identities matter for their development and wellbeing.

### Limitations

There might be some limitations in the study. The students response may have been affected by the context, for instance, the author’s and other students presence. The questionnaires are translated into Norwegian and validated with the help of students who are older than the representative students and may thus have influenced the understanding of the questions. This may explain the low alpha values of some variables. The samples of the studies were recruited students from two school in one city in Norway. Keeping in view the scope of this study, the samples were adequate. However, for future research it would be beneficial to include samples from other countries so as to increase its generalizability and external validity.

## Conclusion

This study investigated the influence of gender on self-concept among high school students enrolled in sport-specialized programs, as well as the connections between self-concept, motivational orientations, and the fulfillment of basic psychological needs.

The findings supported two main hypotheses: male students reported a higher physical self-concept, while no significant gender differences were observed in overall self-concept. Additionally, female students exhibited a stronger emotional self-concept, consistent with existing research on gender differences in emotional awareness and empathy.

Furthermore, the hypothesis suggesting that students with higher physical self-concept would demonstrate higher levels of intrinsic motivation toward sports activities was rejected. Significant associations were instead observed between academic self-concept and intrinsic motivation, as well as between family self-concept and integrated regulation, which represents one of the most autonomous forms of extrinsic motivation.

The findings from this study provide only partial support for the hypothesis that satisfaction of the basic psychological needs autonomy, competence, and relatedness is positively associated with higher self-concept across academic, social, emotional, physical, and family domains. Only academic and family self-concept demonstrated consistent and statistically significant relationships with all three needs.

Social self-concept was associated only with relatedness, Neither physical nor emotional self-concept showed significant associations with any of the basic psychological needs. Overall, the pattern of results suggests that the connection between basic psychological need satisfaction and self-concept is domain-specific rather than universal. While academic and family self-concept appear strongly linked to the fulfillment of autonomy, competence, and relatedness, social, physical, and emotional self-concept show weaker or more selective associations.

## Data Availability

The raw data supporting the conclusions of this article will be made available by the authors, without undue reservation.

## References

[ref1] Alkhateeb H. M. Abushihab E. F. Alkhateeb B. H. Alkhateeb R. H. (2022). Academic self-concept and its relationship to academic achievement among university students. Int. J. Soc. Educ. Sci. 4, 517–528. doi: 10.46328/ijonses.342

[ref2] Ames C. Archer J. (1988). Achievement goals in the classroom: student's learning strategies an motivation processes. J. Educ. Psychol. 80, 260–267. doi: 10.1037/0022-0663.80.3.260

[ref4] Baron A. S. Schmader T. Cvencek D. Meltzoff A. N. (2014). “The gendered self-concept. how implicit gender stereotypes and attitudes shape self-definition,” in Gender and Development, eds. Leman P. J. Tenenbaum H. R. (East Sussex: Psychology Press), 109–132.

[ref5] Chacon-Cuberos R. Serrano-Garcia J. Serrano-Garcia I. Castro-Sanches M. (2025). Self-concept modulates motivation and learning strategies in higher education: comparison according to sex. Educ. Sci. 15, 1–19. doi: 10.3390/educsci15070873

[ref6] Chen W. Sun Y. He Y. (2024). The relationship between parental autonomy support and children’s self-concept in China—the role of basic psychological needs. Behav. Sci. 14:415. doi: 10.3390/bs14050415, 38785906 PMC11117511

[ref7] Deci E. L. Ryan R. M. (1985). Intrinsic Motivation and Self-Determination in Human Nature. New York: Plenum Press.

[ref8] Deci E. L. Ryan R. M. (2002). Handbook of Self-Determination Research. Rochester, NY: The University of Rochester Press.

[ref9] Dimmock J. A. Krause A. E. Rebar A. Jackson B. (2021). Relationships between social interactions, basic psychological needs, and wellbeing during the COVID-19 pandemic. Psychol. Health 37, 457–469. doi: 10.1080/08870446.2021.1921178, 33998909

[ref10] Erdvik I. Haugen T. Ivarsson A. Säfvenbom R. (2019). Development of basic psychological need satisfaction in physical education. Effects of a two-year PE programme. J. Res. Arts Sports Educ. 3, 4–21. doi: 10.23865/jased.v3.1375

[ref11] Flores Kanter P. E. Medrano L. A. Conn H. (2015). Does mood affect self-concept? Analysis through a natural semantic networks based approach. Int. J. Behav. Res. Psychol. 3, 114–120. doi: 10.19070/2332-3000-1500022

[ref12] Fraguela-Vale R. Varela-Garaote L. Carretro-Garcia M. Perlabo-Rubio A. M. (2020). Basic psychological needs, physical self-concept, and physical activity among adolescents: autonomy in focus. Front. Psychol. 20, 1–12. doi: 10.3389/fpsyg.2020.00491 PMC7100532 32265796

[ref13] Garcia F. Martinez I. Balluerka N. Cruise E. Garcia O. F. Serra E. (2018). Validation of the five-factor self-concept questionnaire AF5 in Brazil: testing factor structure and measurement invariance across language (Brazilian and Spanish), gender, and age. Front. Psychol. 9, 1–14. doi: 10.3389/fpsyg.2018.02250, 30515120 PMC6256062

[ref14] Gore J. Cross S. E. (2011). Defining and measuring self-concept change. Psychol. Stud. 56, 135–141. doi: 10.1007/s12646-011-0067-0

[ref15] Grau P. Perez D. Gasco V. Calabuig F. (2014). Self-concept in preadolescence: a brief version of AF5 scale. Motriz Rev. Educ. Fis. 20, 151–157. doi: 10.1590/S1980-65742014000200004

[ref16] Hair J. Anderson R. Babin B. Black W. (2018). Multivariate Data Analysis. 8th Edn New Jersey: Cengage Learning EMEA.

[ref17] Jiang C. Razak N. Raysyid N. Cheng H. (2025). Parents’ sports-related behaviors, self-concept of ability, interest and organized after-school sports activities participation among Chinese elementary school children. Front. Psychol. 16. doi: 10.3389/fpsyg.2025.1581296 PMC12066595 40357471

[ref18] Johansen M. Eliassen S. Jeno L. M. (2023). The bright and dark side of autonomy: how autonomy support and thwarting relate to student motivation and academic functioning. Front. Educ. 8, 1–13. doi: 10.3389/feduc.2023.1153647

[ref19] Khalaila R. (2015). The relationship between academic self-concept, intrinsic motivation, test anxiety, and academic achievement among students: mediating and moderating effects. Nurse Educ. Today 35, 432–438. doi: 10.1016/j.nedt.2014.11.001, 25466798

[ref20] Lavrakas P. (2008). Encyclopedia of Survey Research Methods. 1st Edn. Thousand Oaks: SAGE. doi: 10.4135/9781412963947

[ref21] Löffler C. Greitemeyer T. (2021). Are women the more empathetic gender? The effects of gender role expectations. Curr. Psychol. 42, 220–231. doi: 10.1007/s12144-020-01260-8

[ref22] Lohbeck A. von Keitz P. Hohmann A. Daseking M. (2021). Children's physical self-concept, motivation, and physical performance: does physical self-concept or motivation play a mediating role? Front. Psychol. 12, 1–11. doi: 10.3389/fpsyg.2021.669936, 33995228 PMC8121452

[ref23] Mallett C. Kawabata M. Newcombe P. (2007). Progressing measurement in sport motivation with the SMS-6: a response to Pelletier, Vallerand, and Sarrazin. Psychol. Sport Exerc. 8, 622–631. doi: 10.1016/j.psychsport.2007.05.001

[ref24] Marcic R. Kobal Grum D. (2011). Gender differences in self-concept and self-esteem components. Stud. Psychol. 53, 373–384.

[ref25] Marsh H. M. Shavelson R. J. (1985). Self-concept: its multifaced, hierarchical structure. Educ. Psychol. 20, 107–123. doi: 10.1207/s15326985ep2003_1

[ref26] Martin-Albo J. Nunez J. L. Dominguez E. Leon J. Tomàs J. M. (2012). Relationships between intrinsic motivation, physical self-concept and satisfaction with life: a longitudinal study. J. Sport Sci. 30, 337–347. doi: 10.1080/02640414.2011.649776, 22243036

[ref27] Martinez-Marin M. Martinez C. Paterna C. (2021). Gendered self-concept and gender as predictors of emotional intelligence: a comparison through of age. Curr. Psychol. 40, 4205–4218. doi: 10.1007/s12144-020-00904-z

[ref28] Nocito M. Malaponte N. Malara E. Mandica G. Mangone A. . (2020). Family functioning, basic needs and psychological well-being. J. Clin. Dev. Psychol. 2, 14–26. doi: 10.6092/2612-4033/0110-2858

[ref29] Özcan M. Cekmecelioglu H. G. Konakay G. (2025). Reflections of self-concept clarity at work: the mediating role of psychological empowerment on the relationship between self-concept clarity and intrinsic motivation. BMC Psychol. 13:470, 1–12. doi: 10.1186/s40359-025-02800-2 40329409 PMC12054189

[ref30] Pavlovic S. Pelemis V. Markovic J. Dimintrijevic M. Badric M. Halasi S. . (2023). The role of motivation and physical self-concept in accomplishing physical activity in primary school children. Sports 11, 1–12. doi: 10.3390/sports11090173 PMC10535512 37755850

[ref31] Pelletier L. Rocchi M. Vallerand R. Deci E. L. Ryan R. (2013). Validation of the revised sport motivation scale (SMS-II). Psychol. Sport Exerc. 14, 329–341. doi: 10.1016/j.psychsport.2012.12.002

[ref32] Pelletier L. Sarrazin P. (2007). The revised six-factor sport motivation scale (Mallett, Kawabata, Newcombe, Otero-Forero, & Jackson, 2007): something old, something new, and something borrowed. Psychol. Sport Exerc. 8, 615–621. doi: 10.1016/j.psychsport.2007.03.006615e621

[ref33] Ratelle C. F. Guay F. Vallerand R. Larose S. Senecal C. (2007). Autonomous, controlled, and amotivated types of academic motivation: a person-oriented analysis. J. Educ. Psychol. 99, 734–746. doi: 10.1037/0022-0663.99.4.734

[ref34] Ryan R. M. Deci E. L. (2000). Self-determination theory and the facilitation of intrinsic motivation, social development, and well-being. Am. Psychol. 55, 68–78. doi: 10.1037/0003-066X.55.1.68, 11392867

[ref35] Ryan R. M. Deci E. L. (2017). Self-Determination Theory: Basic Psychological Needs in Motivation, Development and Wellness. New York: The Guilford Press.

[ref36] Ryan R. M. Deci E. L. (2019). “Brick by brick: the origins, development, and future of self-determination theory,” in Advances in Motivation Science, ed. Elliot A.. 6th ed (Cambridge, Mass: Academic Press), 111–156.

[ref37] Ryan R. M. Deci E. L. (2020). Intrinsic and extrinsic motivation from a self-determination theory perspective: definitions, theory, practices, and future directions. Contemp. Educ. Psychol. 61, 1–31. doi: 10.1016/j.cedpsych.2020.101860

[ref38] Shavelson R. J. Hubner J. J. Stanton G. C. (1976). Self-concept: validation of construct interpretations. Rev. Educ. Res. 46, 407–441. doi: 10.3102/00346543046003407

[ref39] Stake J. E. (1992). Gender differences and similarities in self-concept within everyday life contexts. Psychol. Women Q. 16, 349–363. doi: 10.1111/j.1471-6402.1992.tb00259.x

[ref40] Steinberg O. Kulakow S. Raufelder D. (2024). Academic self-concept, achievement, and goal orientations in different learning environments. Eur. J. Psychol. Educ. 39, 3893–3917. doi: 10.1007/s10212-024-00825-6

[ref41] Vansteenkiste M. Deci E. L. (2003). Competitively contingent rewards and intrinsic motivation: can losers remain motivated? Motiv. Emot. 27, 273–299. doi: 10.1023/A:1026259005264

[ref42] Vansteenkiste M. Ryan R. M. Soenens B. (2020). Basic psychological need theory: advancements, critical themes, and future directions. Motiv. Emot. 44, 1–31. doi: 10.1007/s11031-019-09818-1

[ref43] Vansteenkiste M. Simons J. Soenens B. Lens W. (2004). How to become a persevering exerciser: the importance of providing a clear, future goal in an autonomy-supportive way. J. Sport Exerc. Psychol. 26, 232–249. doi: 10.1123/jsep.26.2.232

[ref44] Vansteenkiste M. Soenens B. Lens W. (2007). “Intrinsic versus extrinsic `goal promotion in exercise and sport, understanding the differential impacts on performance and persistence,” in Intrinsic Motivation and Self-Determination in Exercise and Sport, eds. Hagger M. S. Chatzisarantis N. L. D. (Champaign, IL: Human Kinetics).

[ref45] Vansteenkiste M. Soenens B. Ryan R. (2023). “Basic psychological needs theory: a conceptual and empirical review of key criteria,” in The Oxford Handbook of Self-Determination, ed. Ryan R. M. (New York: Oxford University Press), 84–123.

[ref46] Vlachopoulos S. P. Katartzi E. S. Kontou M. G. (2011). The basic psychological needs in physical education scale. J. Teach. Phys. Educ. 30, 263–280. doi: 10.1123/jtpe.30.3.263

[ref47] Vlachopoulos S. P. Ntoumanis N. Smith A. L. (2010). The basic pshychological needs in exercise scale: translation and evidence for cross-cultural validity. Int. J. Sport Exerc. Psychol. 4, 394–412. doi: 10.1080/1612197X.2010.967160

[ref48] Waghmare R. D. (2018). Gender difference between self-concept. Int. J. Indian Psychol. 6, 48–59. doi: 10.25215/0601.067

[ref49] Wang C. K. J. Hagger M. S. (2023). “Self-determination theory in physical activity contexts,” in Oxford Handbook of Self-Determination, ed. Ryan R. M. (New York: Oxford University Press), 740–759.

[ref50] Wilgenbusch T. Merell K. W. (1999). Gender differences in self-concept among children and adolescents: a meta-analysis of multidimensional studies. Sch Psychol. Q. 14, 101–120. doi: 10.1037/h0089000

[ref51] Wilson P. M. Rodgers W. M. Blanchard C. M. Gessell J. (2003). The relationship between psychological needs, self‐determined motivation, exercise attitudes, and physical fitness 1. J. Appl. Soc. Psychol. 33, 2373–2392. doi: 10.1111/j.1559-1816.2003.tb01890.x

